# Antiviral Activity of Feline BCA2 Is Mainly Dependent on Its Interference With Proviral Transcription Rather Than Degradation of FIV Gag

**DOI:** 10.3389/fmicb.2020.01230

**Published:** 2020-06-11

**Authors:** Meng Qu, Weiran Wang, Weiting Li, Jiaming Cao, Xin Zhang, Chu Wang, Jiaxin Wu, Bin Yu, Haihong Zhang, Hui Wu, Wei Kong, Xianghui Yu

**Affiliations:** ^1^National Engineering Laboratory for AIDS Vaccine, School of Life Sciences, Jilin University, Changchun, China; ^2^Key Laboratory for Molecular Enzymology and Engineering of the Ministry of Education, School of Life Sciences, Jilin University, Changchun, China

**Keywords:** host restriction factor, BCA2, NF-κB, transcription, HIV-1 Gag, FIV Gag

## Abstract

Human BCA2/RNF115/Rabring7 (hBCA2) is a RING type E3 ubiquitin ligase with the ability of autoubiquitination or promoting protein ubiquitination. It also acts as a host restriction factor has BST2-dependent and BST2-independent antiviral activity to inhibit the release of HIV-1. In a previous study, we demonstrated that feline BCA2 (fBCA2) also has E3 ubiquitin ligase activity, although its antiviral mechanism remained unclear. In this study, we showed that fBCA2 can interact with feline BST2 (fBST2) and exhibits an fBST2-independent antiviral function, and the RING domain is necessary for the antiviral activity of fBCA2. fBCA2 could degrade HIV-1 Gag and restrict HIV-1 transcription to counteract HIV-1 but not promote the degradation of HIV-1 through lysosomal. Furthermore, for both fBCA2 and hBCA2, restricting viral transcription is the main anti-FIV mechanism compared to degradation of FIV Gag or promoting viral degradation. Consequently, transcriptional regulation of HIV or FIV by BCA2 should be the primary restriction mechanism, even though the degradation mechanism is different when BCA2 counteracts HIV or FIV. This may be due to BCA2 has a special preference in antiviral mechanism in the transmission of primate or non-primate retroviruses.

## Introduction

Lentiviruses, which are a genus within the Orthoretrovirinae subfamily that belongs to the Retroviridae family, can infect T cells and cause slow disease progression. The domestic cat lineage can be infected by retroviruses such as feline immunodeficiency virus (FIV), feline leukemia virus (FeLV), and feline foamy virus (FFV). Cats also harbor the endogenous RD114 gamma retrovirus (Reeves and O’Brien, 1984; Reeves et al., 1985) and full-length endogenous FeLVs (Roca et al., 2004). FIV shares several relevant features with human immunodeficiency virus type 1 (HIV-1) (Elder et al., 2010) and can lead to high levels of immunostimulation in *Felis catus* and an immunodeficiency syndrome similar to that induced by HIV-1 in humans (Pecon-Slattery et al., 2008; Troyer et al., 2008). Additionally, FIV enters T cells via CXCR4 and CD134 and exhibits similarities with HIV-1 in its genomic structure, propagation mechanism, infection process, and pathogenicity (Tomonaga et al., 1992; Willett et al., 1997a, b; Poeschla and Looney, 1998; Shimojima et al., 2004). Thus, domestic cats are considered as relevant natural animal models for studying acquired immunodeficiency syndrome (AIDS) in humans, as well as the development of potential therapeutic strategies for immune control leading to non-progression (Lehman et al., 2010; Yamamoto et al., 2010; Poeschla, 2011).

Retroviruses such as HIV-1, FIV, and other animal viruses exploit cellular molecules and pathways to ensure virus replication (Goff, 2007). In contrast, humans and other mammals have evolved multiple systems to suppress different stages of the virus life cycle through the actions of innate host cell proteins, generally known as restriction factors (Bieniasz, 2004; Goff, 2004). Several restriction factors have been shown to impact the HIV-1 lifecycle and protect host cells from infection, including cytidine deaminase APOBEC3 family proteins (Sheehy et al., 2002; Peng et al., 2006; Tanaka et al., 2006), tetherin/BST2 (Neil et al., 2007, 2008; Van Damme et al., 2008), TRIM5α/TRIMCyp (Stremlau et al., 2004; Song et al., 2005; Yap et al., 2005), SAMHD-1 (Laguette et al., 2011; Laguette et al., 2012; Lahouassa et al., 2012), MX2 (Goujon et al., 2013; Kane et al., 2013), SERINC proteins (Rosa et al., 2015; Usami et al., 2015), and BCA2 (Miyakawa et al., 2009; Nityanandam and Serra-Moreno, 2014; Colomer-Lluch and Serra-Moreno, 2017). Human BCA2 (hBCA2) was first identified as a co-factor of human BST2 (hBST2) and interacts with the cytoplasmic tail of the protein to promote intracellular accumulation and lysosomal degradation of hBST2-trapped virions (Miyakawa et al., 2009). Subsequent studies showed that hBCA2 has BST2-independent anti-HIV-1 activity (Nityanandam and Serra-Moreno, 2014; Colomer-Lluch and Serra-Moreno, 2017). Human BCA2 prevents the assembly and release of nascent virions by promoting the lysosomal degradation of HIV-1 Gag (Nityanandam and Serra-Moreno, 2014) and significantly restricts HIV-1 transcription by inhibiting the NF-κB pathway (Colomer-Lluch and Serra-Moreno, 2017). While the genome of feline (*Felis catus*) also encodes a BCA2 protein (fBCA2) and has demonstrated autoubiquitination activity (Wang et al., 2017), whether it has antiviral activity is unclear.

In the present study, we investigated the mechanism by which fBCA2 inhibits HIV-1 and FIV. Although feline BST2 (fBST2) has a shorter cytoplasmic region compared to those of other mammalian and non-mammalian homologs (Celestino et al., 2012), fBCA2 could interact with fBST2 and showed fBST2-independent antiviral functions. Notably, fBCA2 could degrade HIV-1 Gag but not completely via the lysosomal pathway, which is partly different from hBCA2, and fBCA2 could restrict HIV-1 transcription by inhibiting the NF-κB pathway. In addition, Both hBCA2 and fBCA2 have the ability to restrict FIV. The main antiviral mechanism is the ability to restrict FIV transcription by inhibiting the NF-κB pathway, compared with the ability to degrade FIV Gag. These observations improve our understanding of the different antiviral mechanisms of BCA2 proteins in different species.

## Results

### Feline BCA2 Exhibits fBST2-Independent Antiviral Activity

**FIGURE 1:**
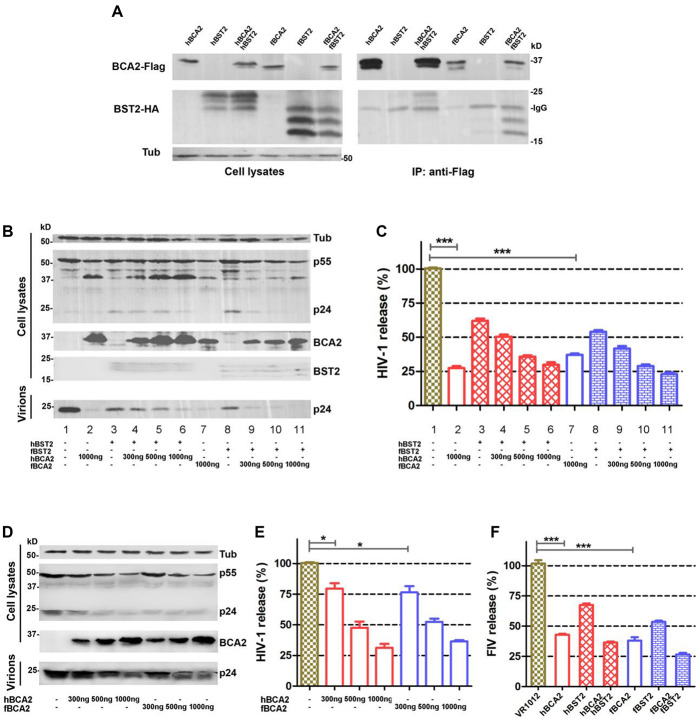
Antiviral activity of fBCA2. **(A)** HEK293T cells were co-transfected with 3 μg of hBCA2-Flag or fBCA2-Flag and 3 μg of hBST2-HA or fBST2-HA. Cell lysates were immunoprecipitated with anti-Flag antibody and protein G, followed by western blotting with anti-HA, anti-Flag, and anti-tubulin antibodies. **(B)** fBST2-HA or hBST2-HA (25 ng) was co-transfected with the proviral plasmid pNL43ΔVpu (1 μg) and fBCA2-Flag (300, 500, or 1000 ng), hBCA2-Flag (300, 500, or 1000 ng), or VR1012 into HEK293T cells. At 48 h post-transfection, the culture supernatants were ultra-centrifuged to concentrate the virus particles. Virions and cell lysates were analyzed by western blotting using anti-p24 antibody to detect viral p24 and intracellular p55Gag proteins, anti-HA to detect the expression of BST2, anti-Flag to detect the expression of BCA2, as well as an antibody against tubulin to assess sample loading. Results of quantitative band scan analysis of the released virions were shown in **(C)**. **(D)** Proviral plasmid pNL43ΔVpu (1 μg) was co-transfected with the fBCA2-Flag (300, 500, or 1000 ng), hBCA2-Flag (300, 500, or 1000 ng), or a VR1012 into HEK293T cells. At 48 h post-transfection, the culture supernatants were ultra-centrifuged to concentrate the virus particles. Virions and cell lysates were analyzed by western blotting using anti-p24, anti-Flag, and anti-tubulin antibodies. Results of quantitative band scan analysis of the released virions were shown in **(E)**. **(F)** FIV-ΔVif-ΔOrfA-ΔEnv-GFP virus plasmid (pFP93, pGINSIN, and pVSV-G) (1 μg) was co-transfected with the fBCA2-Flag 500 ng, hBCA2-Flag 500 ng, and fBST2-HA (25 ng), hBST2-HA (25 ng), or VR1012 into HEK293T cells. At 48 h post-transfection, the supernatants from the producer cells were harvested, clarified and assayed for GFP fluorescence intensity using a fluorescence microplate reader to quantify released virions. The data were presented as the means ± SDs for three independent experiments. Statistical significance was analyzed by using Student’s *t*-test. Significant differences are indicated as follows: *P < 0.05; ***P < 0.001.

Human BCA2 interacts with the cytoplasmic tail of BST2 to promote the intracellular accumulation and lysosomal degradation of BST2-trapped virions (Miyakawa et al., 2009), but it was also found to have BST2-independent anti-HIV-1 activity (Nityanandam and Serra-Moreno, 2014; Colomer-Lluch and Serra-Moreno, 2017). However, studies indicated that fBST2 has a much shorter cytoplasmic region compared to those of other mammalian and non-mammalian homologs (Celestino et al., 2012). In order to verify whether fBCA2 could interacts with fBST2, we performed an immunoprecipitation assay with HEK293T cells transfected with hBCA2-Flag, fBCA2-Flag, and either hBST2-HA or fBST2-HA. Cell lysates were precipitated with an anti-Flag antibody and protein G, followed by western blot analysis with anti-HA and anti-Flag antibodies. The results showed that fBCA2 could interact with fBST2, although fBST2 has a short cytoplasmic tail, which is similar to the interaction of hBCA2 with hBST2 (Figure 1A). To further study the antiviral activity of BCA2, we investigated the effects of these proteins on the release of HIV-1 in the presence and absence of hBST2 or fBST2. HEK293T cells were co-transfected with NL43ΔVpu, along with either hBST2-HA or fBST2-HA, hBCA2-Flag, or fBCA2-Flag and VR1012 (empty vector). The cell lysates and released virions were then analyzed by western blotting, and tubulin was detected as a loading control (Figure 1B). The HIV-1 viral particles released from cells only expressing hBST2-HA or fBST2-HA showed a significant decrease (∼40%) (Figure 1C, lanes 3 and 8). When HEK293T cells expressed both fBST2-HA and different amounts of fBCA2-Flag, the released HIV-1 virion was reduced in a dose-dependent manner (Figure 1C, lanes 9, 10, and 11); the same results were observed for hBST2-HA and hBCA2-Flag (Figure 1C, lanes 4, 5, and 6). In contrast, cells that only expressed hBCA2-Flag and fBCA2-Flag showed significant anti-HIV-1 activity (Figure 1C, lane 2 and 7). The results showed that both BST2 and BCA2 exhibited inhibitory effect on HIV-1 release. Furthermore, when HEK293T cells were expressed fBCA2-Flag or hBCA2-Flag without BST2-HA, the released HIV-1 virions were reduced in a dose-dependent manner (Figures 1D,E). Meanwhile, combined with the quantification analysis of Figures 1C,E, we found that cells expressing fBCA2-Flag (300 ng) alone or with fBST2-HA showed a nearly 25 or 60% decrease in HIV-1 viral particles release, respectively (Figure 1E, lane 5 and Figure 1C, lane 9), indicating that fBCA2 could cooperate with fBST2 in viral restriction when fBCA2 protein level is low. The same results were observed for hBCA2-Flag and hBST2-HA (Figure 1E, lane 2 and Figure 1C, lane 4), which means that BCA2 not only has independent anti-HIV ability but also could work together with BST2. In addition, hBCA2-Flag (1000 ng) has similar antiviral ability in the absence and presence of hBST2-HA (Figure 1E, lane 4 and Figure 1C, lanes 2 and 6), which means that when hBCA2 protein level is high, it plays the main role in antiviral activity. Therefore, BCA2 exhibits BST2-independent anti-HIV-1 activity. In addition, HEK293T cells were co-transfected with FIV-ΔVif-ΔOrfA-ΔEnv-GFP virus plasmid (pFP93, pGINSIN, and pVSV-G), along with either hBST2-HA or fBST2-HA, hBCA2-Flag, or fBCA2-Flag and VR1012. The supernatants from the producer cells were harvested, clarified and assayed for GFP fluorescence intensity using a fluorescence microplate reader to quantify released virions. Similar to the decrease in HIV-1 release, both BCA2 and BST2 had the ability to reduce the release of FIV virions (Figure 1F). The FIV particles released from cells only expressing hBCA2-Flag or fBCA2-Flag showed a significant decrease (∼60%) (Figure 1F, lanes 2 and 4). Consequently, fBCA2 can cooperate with fBST2 in decreasing viral release but also has fBST2-independent anti-HIV-1 and anti-FIV activity.

### Feline BCA2 Hardly Promotes Lysosomal Degradation of HIV-1

**FIGURE 2:**
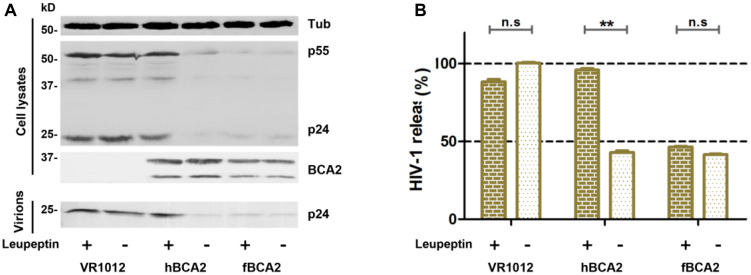
Feline BCA2 cannot promote lysosomal degradation of HIV-1. **(A)** HEK293T cells transfected with 1 μg of pNL43 and 500 ng of hBCA2-Flag, fBCA2-Flag, or VR1012 were treated with 20 μM lysosomal inhibitor leupeptin or DMSO as a control. Leupeptin was added to the medium at 24 h before harvesting. Cell lysates and supernatants were then analyzed by western blotting using anti-p24, anti-Flag, and anti-tubulin antibodies. Results of quantitative band scan analysis of the released virions were shown in **(B)**. The data were presented as the means ± SDs for three independent experiments. Statistical significance was analyzed by using Student’s *t*-test. Significant differences are indicated as follows: n.s > 0.05; **P < 0.01.

hBCA2 can interact with Rab7 and enhance the targeting of HIV-1 virions for lysosomal degradation but not proteasomal degradation (Mizuno et al., 2003; Miyakawa et al., 2009; Nityanandam and Serra-Moreno, 2014). To investigate whether fBCA2 also has this degradation mechanism, the antiviral effects of fBCA2 were analyzed in the presence of lysosomal inhibitor (Leupeptin) or proteasomal inhibitor (MG132). HEK293T cells were co-transfected with HIV-1 pNL43 and hBCA2-Flag, fBCA2-Flag, or VR1012. Twenty-four hours post-transfection, either DMSO, Leupeptin or MG132 was added to the medium. The lysates and released capsid proteins of cells transfected with HIV-1 proviral DNA were then analyzed by western blotting, and tubulin was detected as a loading control (Figure 2A). As expected, transfection with hBCA2-Flag or fBCA2-Flag resulted in a threefold reduction in the number of virus particles present in the culture supernatant compared to transfections with VR1012 (Figure 2B, lanes 2, 4, and 6). Addition of Leupeptin to cells expressing hBCA2-Flag resulted in an increase in virus release compared to cells expressing hBCA2-Flag and treated with DMSO (Figure 2B, lanes 3 and 4), which is consistent with previous reports (Miyakawa et al., 2009; Nityanandam and Serra-Moreno, 2014). However, addition of Leupeptin to cells expressing fBCA2-Flag had little effect on virus release (Figure 2B, lanes 5 and 6), and fBCA2 did not promote the degradation of HIV-1 through lysosome or proteasome (Supplementary Figure S1), suggesting that fBCA2 utilizes an antiviral pathway that is not exactly the same as that used by hBCA2.

### C229C232(C228C231) in the RING Domain of Feline and Human BCA2 Are Essential for Anti-HIV-1 Activity

**FIGURE 3:**
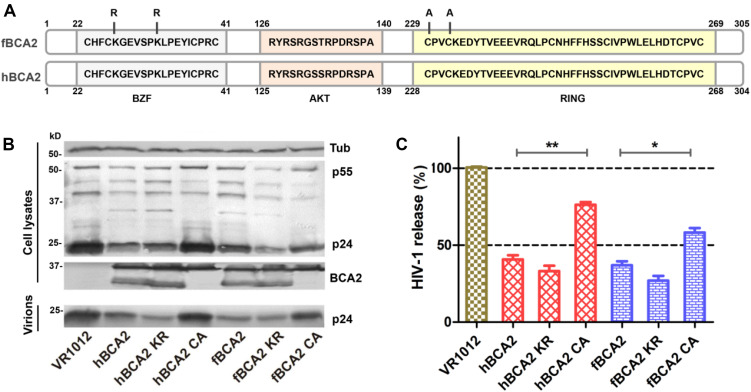
Anti-HIV-1 function of fBCA2 mutants. **(A)** Schematic representation of human and feline BCA2 with the zinc-finger domain (BZF), AKT-phosphorylation sites, and RING domain. Key residues of BCA2 mutants (hBCA2 KR-Flag, fBCA2 KR-Flag, hBCA2 CA-Flag, and fBCA2 CA-Flag) are indicated. **(B)** HEK293T cells were co-transfected with 500 ng of different variants of BCA2 or VR1012 and 1 μg of the proviral plasmid pNL43. After 48 h, the culture supernatants were ultracentrifuged to collect the virus particles. Virions and cell lysates were analyzed by western blotting using anti-p24 antibody, anti-tubulin antibody, and anti-Flag antibody. Results of quantitative band scan analysis of the released virions were shown in **(C)**. The data were presented as the means ± SDs for three independent experiments. Statistical significance was analyzed by using Student’s *t*-test. Significant differences are indicated as follows: *P < 0.05; **P < 0.01.

Human BCA2 contains a BZF domain in the N-terminal region of the protein, which has been shown to interact with ubiquitin, and K26 and K32 lysine mutations were found to abrogate autoubiquitination activity (Amemiya et al., 2008). The RING domain in the C-terminus has been shown to interact with E2, which confers autoubiquitination activity, while the C228 and C231 cysteine mutation prevents the interaction with E2 (Hu and Fearon, 1999; Burger et al., 2005; Amemiya et al., 2008; Wang et al., 2017). As the E3 ligase activity of BCA2 is dispensable for BST2-dependent restriction but required for BST2-independent restriction (Nityanandam and Serra-Moreno, 2014), we constructed mutations at certain key residues in BCA2. Mutants at ubiquitination sites (K26 K32) were designated as hBCA2 KR or fBCA2 KR, and a mutant in the E3 catalytic domain (C229 C232) was designated as hBCA2 CA or fBCA2 CA (Figure 3A; Amemiya et al., 2008). The wild-type BCA2 as well as mutants were analyzed to determine if they retained their anti-HIV-1 activity. First, HEK293T cells were co-transfected with wild-type or different variants of BCA2 or VR1012 and pNL43. The cell lysates and released capsid proteins were then analyzed by western blotting, and tubulin was detected as a loading control (Figure 3B). The release of HIV-1 viral particles from cells expressing hBCA2-Flag (Figure 3C, lane 2) or fBCA2-Flag (Figure 3C, lane 5) was significantly decreased. Meanwhile, the anti-HIV-1 particle release of BZF mutants hBCA2 KR/fBCA2 KR is similar with wild-type hBCA2/fBCA2 (Figure 3C, lanes 3 and 6). In contrast, the RING domain mutants hBCA2 CA-Flag and fBCA2 CA-Flag displayed attenuation in the efficiency of anti-HIV-1 particle release compared to that of wild-type BCA2 (Figure 3C, lanes 4 and 7). The antiviral activities of hBCA2, hBCA2 KR, and hBCA2 CA were consistent with previously reported results (Miyakawa et al., 2009; Nityanandam and Serra-Moreno, 2014). Feline BCA2 have similar antiviral domain to human BCA2.

### BCA2 RING Domain Mutants Cannot Induce HIV-1 Gag Degradation

**FIGURE 4:**
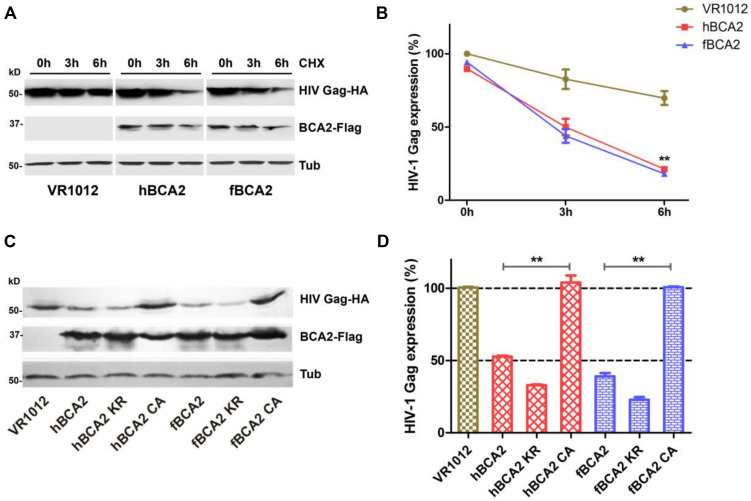
Feline and human BCA2 promote degradation of HIV-1 Gag. **(A)** HEK293T cells were co-transfected with HIV Gag-HA (1000 ng) and hBCA2 (300 ng), fBCA2 (300 ng), or VR1012. At 40 h after transfection, cells were treated with CHX (200 μg/mL) for different time. The cells were harvested and analyzed by western blotting with anti-HA, anti-Flag, and anti-tubulin antibodies. Results of quantitative band scan analysis of HIV Gag were shown in **(B)**. **(C)** HEK293T cells were co-transfected with 500 ng of HIV Gag-HA and 500 ng of different variants of BCA2 or VR1012. The cells were harvested at 48 h after transfection and analyzed by western blotting with anti-HA, anti-Flag, and anti-tubulin antibodies. Results of quantitative band scan analysis of HIV Gag were shown in **(D)**. The data were presented as the means ± SDs for three independent experiments. Statistical significance was analyzed by using Student’s *t*-test. Significant differences are indicated as follows: **P < 0.01.

Promotion of degradation of HIV-1 Gag is one of the anti-HIV-1 function of hBCA2. Next, we asked whether fBCA2 has the same ability. To test this, HEK293T cells were co-transfected with HIV-1 Gag-HA plasmid along with hBCA2, fBCA2, or VR1012. The cells were harvested, and we examined the HIV-1 Gag protein level at different time points after addition of cycloheximide (CHX), which blocks protein synthesis. The results showed that BCA2 significantly increased degradation rate of the HIV-1 Gag over time (Figures 4A,B). Meanwhile, the RING finger domain is important for inducing Gag protein degradation (Nityanandam and Serra-Moreno, 2014). fBCA2 have a RING finger domain similar to that of hBCA2, which interact with E2 and confers autoubiquitination activity (Hu and Fearon, 1999; Burger et al., 2005; Amemiya et al., 2008; Wang et al., 2017). To investigate whether fBCA2 can mediate degradation of HIV-1 Gag and whether the RING domain plays a key role in this process, HEK293T cells were co-transfected with HIV-1 Gag-HA plasmid along with wild-type or different variants of BCA2 or VR1012. Forty-eight hours post-transfection, the cells were harvested and analyzed by western blotting, with tubulin detected as a loading control (Figure 4C). Consistent with previous results (Nityanandam and Serra-Moreno, 2014), hBCA2 and hBCA2 KR degraded HIV-1 Gag (Figure 4D, lanes 2 and 3), while hBCA2 CA completely lost this function (Figure 4D, lane 4). Additionally, fBCA2 showed the same results as hBCA2. The wild-type fBCA2 and ubiquitination site mutant fBCA2 KR effectively degraded HIV-1 Gag (Figure 4D, lanes 5 and 6), while the RING domain mutant fBCA2 CA could not induce degradation of HIV-1 Gag (Figure 4D, lane 7).

### fBCA2 and hBCA2 Restrict HIV-1 Transcription by Inhibiting NF-κB

**FIGURE 5:**
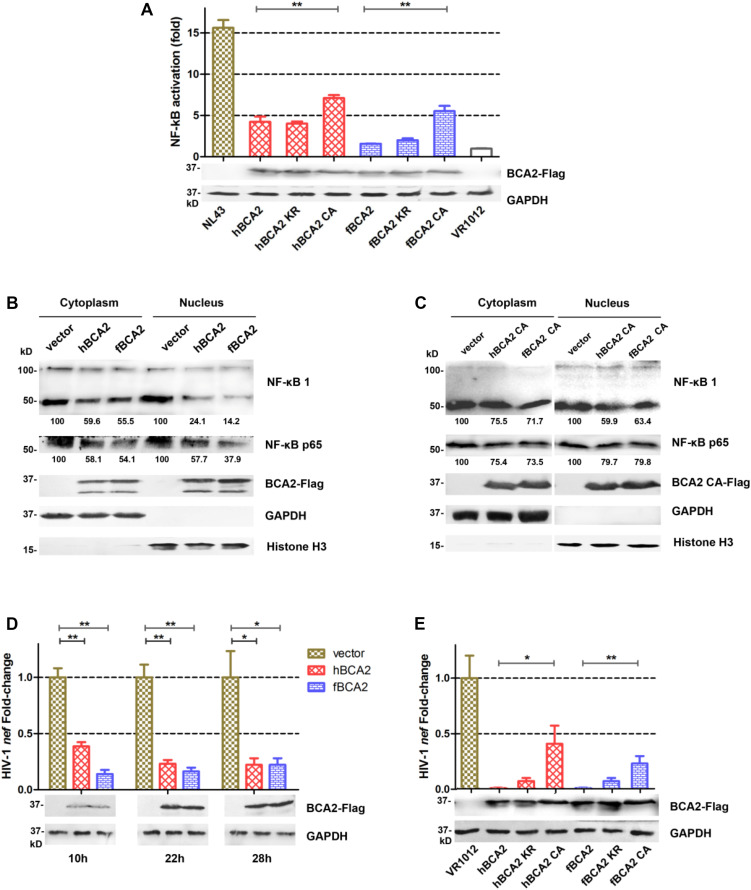
Feline and human BCA2 restrict HIV-1 transcription by preventing HIV-1-induced activation of NF-κB. **(A)** HEK293T cells were co-transfected with 250 ng different variants of BCA2 along with 250 ng of pNF-κB-Luc reporter plasmid and 1 μg of pNL43. After 48 h, the cells were lysed and analyzed for luciferase activity and by western blotting with anti-Flag, and anti-tubulin antibodies. **(B,C)** Five million HEK293T cells were transfected with 5 μg of VR1012, hBCA2, fBCA2, hBCA2 CA, or fBCA2 CA. After 48 h, cytoplasmic and nuclear fractions were separated and analyzed by western blotting to evaluate NF-κB 1(p105/p50), NF-κB p65, BCA2, GAPDH, and Histone H3 in each fraction. Results of quantitative band scan analysis of NF-κB p50/p65 were shown below, and the percentage of VR1012 quantifications are set to 100%. **(D)** HEK293T cells were co-transfected with 1 μg of pNL43, 500 ng of hBCA2-Flag, fBCA2-Flag, or VR1012, and the levels of *nef* RNA were analyzed at 10, 22, and 28 h by RT-qPCR after transfection, and the cell lysates were analyzed by western blotting with anti-Flag, and anti-tubulin antibodies. **(E)** HEK293T cells were co-transfected with 1 μg of pNL43, 500 ng of different variants of BCA2 or VR1012, and the levels of *nef* RNA were analyzed 48 h later by RT-qPCR, and the cell lysates were analyzed by western blotting with anti-Flag, and anti-tubulin antibodies. The data were presented as the means ± SDs for three independent experiments. Statistical significance was analyzed by using Student’s *t*-test. Significant differences are indicated as follows: *P < 0.05; **P < 0.01.

hBCA2 CA and fBCA2 CA could not degrade HIV-1 Gag (Figure 4C) but retained partial anti-HIV-1 activity (Figure 3B), indicating that BCA2 has other anti-HIV-1 mechanism. HIV-1 and other primate lentiviruses have been known to induce NF-κB cascades to facilitate proviral transcription, as they contain NF-κB responsive elements in the regulatory regions of their LTRs (Nabel and Baltimore, 1987; Regier and Desrosiers, 1990). Additionally, inhibiting the NF-κB pathway can significantly restrict HIV-1 transcription (Colomer-Lluch and Serra-Moreno, 2017). To explore whether fBCA2 can prevent HIV-1-induced activation of NF-κB and further restrict HIV-1 transcription, we performed the following experiments. First, we verified the inhibition of fBCA2 on NF-κB activation. HEK293T cells were co-transfected with an expression plasmid for each BCA2 variant or VR1012 along with the pNF-κB-Luc reporter plasmid and pNL43. After 48 h, the cells were lysed and analyzed for luciferase activity (Figure 5A). Whereas NF-κB activation with HIV-1 was increased by approximately 15-fold in the absence of hBCA2 or fBCA2 (Figure 5A, Lane 1, Lane 8), expression of hBCA2 or fBCA2 resulted in inhibition of this cascade, indicating that both hBCA2 and fBCA2 can prevent HIV-1-dependent activation of NF-κB. The inhibition of BZF mutants hBCA2 KR/fBCA2 KR is similar with wild-type hBCA2/fBCA2, and NF-κB was also inhibited by RING mutants hBCA2 CA/fBCA2 CA, but lower than WT (Figure 5A). Additionally, the nuclear and cytoplasmic levels of NF-κB monomers were examined. HEK293T cells were transfected with the VR1012, hBCA2, or fBCA2. Forty-eight hours later, cell lysates were differently fractionated to obtain cytoplasmic and nuclear extracts. Consistent with the previous results, the levels of NF-κB 1 (p100/p50) and NF-κB p65 in nuclear fraction were significantly reduced in the cells expressing hBCA2 and fBCA2. Moreover, similar reductions were found in the cytoplasmic fraction, which means that in addition to blocking nuclear localization of p50/p65 heterodimer to inhibit the nuclear transport of NF-κB, hBCA2, and fBCA2 may also lead to the degradation of p50/p65 in cytoplasm and consequently a reduction in nucleus (Figure 5B). Meanwhile, the level of NF-κB 1 (p100/p50) and NF-κB p65 were reduced by BCA2 CA mutants but the percentage of reductions were lower than that in the presence of the WT BCA2 proteins (Figure 5C).

To assess whether fBCA2 has detrimental effects on the transcriptional capacity of HIV-1, the amount of *nef*-containing transcript levels was measured. The HIV-1 *nef*-specific qPCR primers were designed as previously described (Colomer-Lluch and Serra-Moreno, 2017). HEK293T cells were co-transfected with VR1012, hBCA2, or fBCA2 along with pNL43. We detected the amount of HIV-1 *nef* mRNA at 10, 22, and 28 h post-transfection and observed the transcription levels of HIV-1. We found that hBCA2 and fBCA2 reduced the transcription of HIV-1 at early time points. Remarkably, cells overexpressing hBCA2-Flag showed a 2.6-fold decrease in HIV-1 transcription at only 10 h after provirus transfection, as well as 4.3- and 4.5-fold reductions at 22 and 28 h, respectively (Figure 5D). Meanwhile, fBCA2 also showed a 1. 6-, 5. 2-, and 4.5-fold decrease in HIV-1 transcription at 10, 22, and 28 h post-transfection, respectively (Figure 5D). Thus, both hBCA2 and fBCA2 negatively affected HIV-1 provirus transcription, while viruses from cells transfected with pNL43 and VR1012 were properly transcribed. Simultaneously, BCA2 mutants were examined for their impact on HIV-1 provirus transcription. HEK293T cells were transfected with wild-type or different variants of BCA2 or VR1012 along with pNL43. To maximize the difference, the amount of *nef* -containing transcripts was measured at 48 h after transfection. We found that cells expressing hBCA2 and fBCA2 showed a nearly 100-fold decrease in HIV-1 transcription, and those expressing hBCA2 KR and fBCA2 KR showed relatively weaker inhibition (14-fold); cells transfected with hBCA2 CA or fBCA2 CA showed a 2.5- or 4.3-fold reduction, respectively (Figure 5E). Therefore, RING domain mutants (hBCA2 CA/fBCA2 CA) lost the function of degradation of HIV-1 Gag (Figure 4C), but retained the ability to inhibit NF-κB activation and further inhibit HIV-1 transcription (Figure 5).

### Antiviral Activity Against FIV of BCA2 Is Mainly Due to Restrict Transcription by Preventing FIV-Induced Activation of NF-κB

**FIGURE 6:**
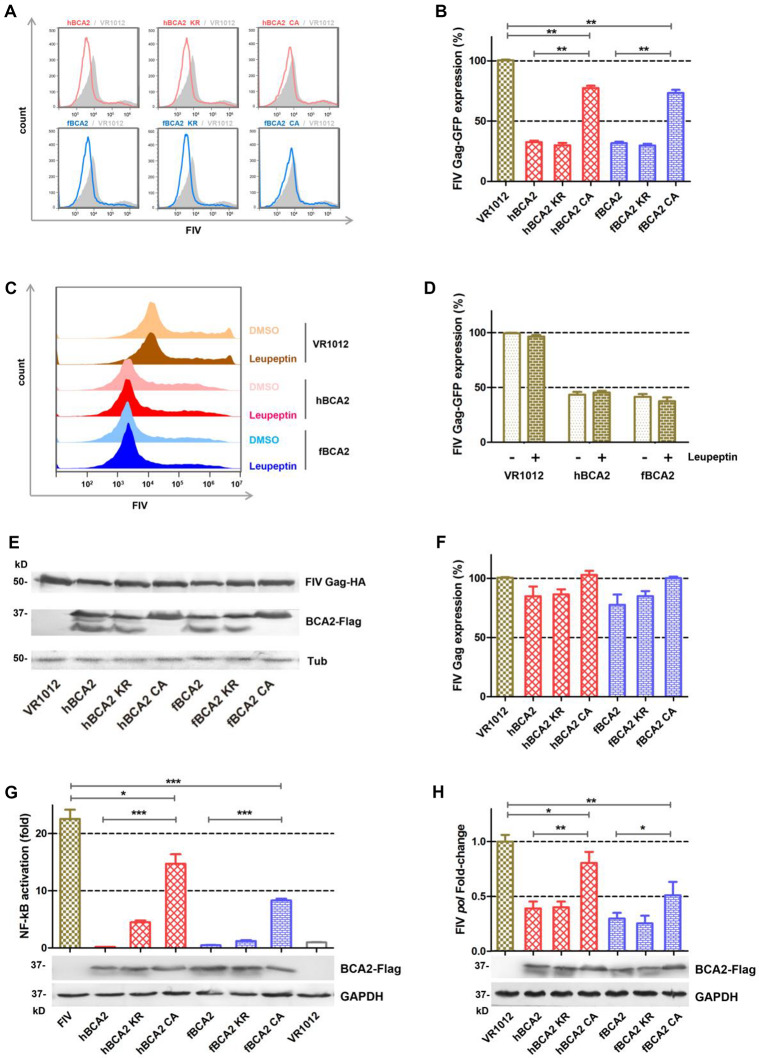
Anti-FIV- functions of fBCA2 and hBCA2. **(A)** VR1012, different variants of BCA2 (500 ng each) was co-transfected with 1 μg of FIV-ΔVif-ΔOrfA-ΔEnv-GFP reporter virus plasmid (pFP93: pGINSIN: pVSV-G = 3:3:1) in HEK293T cells. Forty-eight hours later, the cells were harvested and analyzed for fluorescence intensity by flow cytometry. Mean fluorescent intensity values in panel A are shown in **(B)**. **(C)** HEK293T cells transfected with 1 μg of FIV-ΔVif-ΔOrfA-ΔEnv-GFP reporter virus plasmid (pFP93: pGINSIN: pVSV-G = 3:3:1) and 500 ng of hBCA2-Flag, fBCA2-Flag or VR1012 were treated with 20μM lysosomal inhibitor leupeptin or DMSO as a control. Leupeptin was added to the medium at 24 h before harvest. Cells were harvested and analyzed for fluorescence intensity by flow cytometry. Mean fluorescent intensity values are shown in **(D)**. **(E)** HEK293T cells were co-transfected with 500 ng of FIV Gag-HA and 1 μg of VR1012 or different variants of BCA2. The cells were harvested at 48 h after transfection and analyzed by western blotting with anti-HA, anti-Flag, and anti-tubulin antibodies. Results of quantitative band scan analysis of HIV Gag were shown in **(F)**. **(G)** HEK293T cells were co-transfected with 250 ng of expression plasmid for each variant BCA2 along with 250 ng of pNF-κB-Luc reporter plasmid and 1 μg of FIV-ΔVif-ΔOrfA-ΔEnv-GFP reporter virus plasmid (pFP93: pGINSIN: pVSV-G = 3:3:1). After 48 h, the cells were lysed and analyzed for luciferase activity, and the cell lysates were analyzed by western blotting with anti-Flag, and anti-tubulin antibodies. **(H)** HEK293T cells were co-transfected with VR1012 or different variants of BCA2 along with FIV-14, and the level of *pol* mRNA was analyzed 48 h later by RT-qPCR, and the cell lysates were analyzed by western blotting with anti-Flag, and anti-tubulin antibodies. The data were presented as the means ± SDs for three independent experiments. Statistical significance was analyzed by using Student’s *t*-test. Significant differences are indicated as follows: *P < 0.05; **P < 0.01; ***P < 0.001.

HEK293T cells were co-transfected with different BCA2 plasmids or VR1012 and FIV-ΔVif-ΔOrfA-ΔEnv-GFP virus plasmid (pFP93, pGINSIN, and pVSV-G) to determine if they retained anti-FIV pseudovirus activity (Figure 6A). The cells were analyzed by flow cytometry to assess FIV Gag-GFP protein expression. Similar to the inhibition of HIV-1, hBCA2-Flag, hBCA2 KR-Flag, fBCA2-Flag, and fBCA2 KR-Flag effectively reduced the level of FIV Gag protein (∼70%) (Figure 6B). The two RING domain mutants, hBCA2 CA-Flag and fBCA2 CA-Flag, showed FIV Gag changes (∼25% decrease) compared to that of hBCA2 or fBCA2 (Figure 6B, lanes 4 and 7). Along with the anti-HIV-1 function, these results indicate that the RING domain cysteine mutants negatively impacted the antiviral function of BCA2, while BCA2 with mutated BZF domain lysine residues maintained its antiviral activity.

We next examined if BCA2 could induce the lysosomal degradation of FIV pseudovirus. After transfection with the FIV-ΔVif-ΔOrfA-ΔEnv-GFP reporter virus plasmid and treatment with 20 μM Leupeptin or DMSO, the cells were analyzed by flow cytometry to assess FIV Gag-GFP protein expression (Figure 6C). The FIV Gag-GFP protein expression in cells transfected with hBCA2 or fBCA2 was significantly decreased (∼60%) compared to in cells only transfected with VR1012 (Figure 6D, lanes 3 and 5). The antiviral effect of hBCA2 or fBCA2 on FIV Gag-GFP expression was not affected by addition of Leupeptin (Figure 6D, lanes 4 and 6).

Both hBCA2 and fBCA2 promoted the degradation of HIV-1 Gag and inhibited HIV-1 transcription by preventing the activation of NF-κB (36). The following experiments were performed to verify the mechanism of BCA2 counteracting FIV. First, HEK293T cells were co-transfected with different BCA2 plasmids or VR1012 and FIV Gag-HA. The cell lysates were analyzed by western blotting, and tubulin was detected as a loading control, hBCA2 and fBCA2 only showed a very weak ability to degrade FIV Gag (Figures 6E,F).

HIV-1 and other primate lentiviruses contain NF-κB responsive elements to induce NF-κB cascades to facilitate proviral transcription (Nabel and Baltimore, 1987; Regier and Desrosiers, 1990). To investigate the inhibitory effect of BCA2 on FIV-induced NF-κB activation, HEK293T cells were co-transfected with hBCA2-Flag, fBCA2-Flag, different BCA2 variants, or VR1012 along with the pNF-κB-Luc reporter plasmid and FIV-ΔVif-ΔOrfA-ΔEnv-GFP virus plasmid. 48 h later, the cells were lysed and analyzed for luciferase activity (Figure 6G). Like HIV-1, the FIV contains NF-κB responsive elements and led to a greater than 20-fold induction in NF-κB activity in the absence of BCA2 (Figure 6G, Lanes 1 and 8). wild-type hBCA2/fBCA2 and BZF mutants hBCA2 KR/fBCA2 KR showed strong inhibitory effects on FIV activation of NF-κB. FIV activation of NF-κB was also inhibited by RING mutants hBCA2 CA/fBCA2 CA, but lower than WT. (Figure 6G). To assess whether BCA2 reduces FIV by inhibiting the NF-κB pathway and then restricting proviral transcription, the account of *pol*-containing transcriptions was measured. The FIV-*pol*-specific qPCR primers were designed as previously described (Bisset et al., 2001). HEK293T cells were co-transfected with the VR1012 or different BCA2 variants along with FIV-14. The amount of *pol*-containing transcription was measured at 48 h after transfection. The results showed that cells expressing hBCA2 and hBCA2 KR or fBCA2 and fBCA2 KR showed 2.5- and 3.5-fold decreases in FIV transcription, respectively, while expressing hBCA2 CA and fBCA2 CA showed relatively weaker inhibition (Figure 6H). Therefore, the main anti-FIV mechanism of BCA2 is to restrict proviral transcription by inhibiting virus-induced activation of NF-κB.

## Discussion

Human BCA2 is a RING finger protein possessing ubiquitination ligase activity and first identified in breast cancer cells (Burger et al., 1996, 1998, 2005). Human BCA2 contains a BZF domain in the N-terminal region of the protein which interacts with ubiquitin, and K26 and K32 lysine mutations abrogate its autoubiquitination activity (Amemiya et al., 2008). The RING domain in the C-terminus interacts with E2 (UbcH5b) to confer autoubiquitination activity, while the C228 and C231 cysteine mutants do not interact with E2 (Hu and Fearon, 1999; Burger et al., 2005; Amemiya et al., 2008). Human BCA2 is also an E3 ligase that interacts with the only SUMO conjugating enzyme, Ubc9. BCA2 can therefore function as an E3 ligase in the sumoylation of various transcription factors (Burger et al., 2010). It was first demonstrated that hBCA2 could enhance hBST2 restriction by targeting trapped HIV-1 virions for lysosomal degradation (Miyakawa et al., 2009) and promotes retroviral Gag protein degradation in a BST2-independent manner. Furthermore, a recent report showed that hBCA2 significantly restricted HIV-1 transcription by inhibiting the NF-κB pathway, which would account for most of the antiviral activity of BCA2 (Colomer-Lluch and Serra-Moreno, 2017). An intact RING finger domain is required for promoting retroviral Gag protein degradation (Nityanandam and Serra-Moreno, 2014), and it is also necessary for the catalytic activity of hBCA2 to block NF-κB responses to restrict HIV-1 transcription (Colomer-Lluch and Serra-Moreno, 2017).

The fBCA2 (MF327275) transcript is 918 base pairs in length with 92.6% consensus positions and identity positions, and encodes a protein of 305 amino acids with 96.7% of consensus positions and 93.1% identity positions compared to its human counterpart. Our results showed that fBCA2 also has the same fBST-2-independent antiviral function as hBCA2. Similarities were observed between fBCA2 and hBCA2 in the mechanism of inhibiting virus release. Meanwhile, the antiviral functional domain of both fBCA2 and hBCA2 are related to their E3 ubiquitin ligase function. The wild-type hBCA2/fBCA2 and BZF mutant hBCA2/fBCA2 KR showed significant antiviral activities, while the RING finger domain mutant hBCA2/fBCA2 CA only retained relatively weak antiviral effect (Figures 3, 6), which indicated that the RING domain is necessary for the antiviral activity of hBCA2/fBCA2. In addition, the RING domain at the C-terminus of both hBCA2 and fBCA2 was also found to be essential for autoubiquitination (Wang et al., 2017).

However, the antiviral mechanism of fBCA2 is not the same as that of hBCA2. Firstly, hBCA2 was found to promote the lysosomal degradation of HIV-1 (Nityanandam and Serra-Moreno, 2014), but fBCA2 showed hardly mediation of the degradation of HIV-1 virus particles through the lysosomal pathway (Figure 2). Meanwhile, fBCA2 showed minimal mediation of the degradation of FIV pseudovirus through the lysosomal pathway; and even there is interaction between hBCA2 and Rab7 (Nityanandam and Serra-Moreno, 2014), which can induce lysosomal degradation of HIV-1, hBCA2 still lost the function of lysosomal degradation of FIV (Figure 6C). It means that the induction of virus particle degradation through the lysosomal pathway may not be the main mechanism of BCA2 protein antiviral function. Secondly, the inhibitory mechanism of BCA2 on FIV differed from that against HIV-1. Our data showed that hBCA2 and fBCA2 are capable of degrading HIV-1 Gag but not FIV Gag (Figures 4, 6). However, hBCA2 and fBCA2 still limited the transcription of FIV by inhibiting the activation of NF-κB induced by FIV (Figures 6G,H). These results suggest that the anti-FIV activity of fBCA2 is mostly dependent on its interference with proviral transcription rather than by degradation of Gag or targeting viral degradation through the lysosomal pathway.

Meanwhile, hBCA2/fBCA2 CA mutants could not degrade HIV-1 Gag, but they still showed anti-HIV-1 activity (Figure 3B). Our findings indicate that antiviral activity of BCA CA mutants occurs through inhibition of the NF-κB pathway to restrict HIV-1 transcription. Therefore, the main antiviral mechanism of fBCA2, similarly, to hBCA2 (Colomer-Lluch and Serra-Moreno, 2017), is the ability to restrict FIV transcription by inhibiting the NF-κB pathway compared to the ability to degrade FIV Gag. Moreover, the results showed that the hBCA2/fBCA2 CA mutants impaired the E3 ligase activity, but it still had the ability to restrict HIV-1/FIV transcription. This is different from previous studies which found that BCA2 promotes the SUMOylation of UBC9 to prevent NF-κB activation (Colomer-Lluch and Serra-Moreno, 2017), suggesting that hBCA2 and fBCA2-mediated inhibition of NF-κB is not entirely dependent on the enzymatic activity of BCA2. Other potential mechanisms mediated by BCA2 may still await further exploration.

Indeed, many HIV-1 antiviral factors are involved in the NF-κB innate signaling pathway by mediating NF-κB-dependent immune responses (i.e., TRIM5α, Tetherin), or responding to NF-κB (i.e., APOBEC3G), or controlling viral infections and inflammatory diseases (i.e., SAMHD1) (Pauli et al., 2009; Pertel et al., 2011; Galao et al., 2012, 2014; Tokarev et al., 2013; Uchil et al., 2013; Chen et al., 2018). NF-κB activation enhances the transcription activity of HIV-1, which is critical for the replication of HIV-1. Therefore, BCA2 restriction of proviral transcription by inhibiting the NF-κB pathway is important for its antiviral mechanism.

## Conclusion

In conclusion, BCA2 is conserved among human and cats which also carry their own lentiviruses (human and feline immunodeficiency viruses). Our findings suggest that BCA2 restricts proviral transcription by inhibiting the NF-κB pathway among human and feline immunodeficiency viruses, while targeting of trapped HIV-1 virions for lysosomal degradation and promoting retroviral Gag protein ubiquitination was significantly exist in human immunodeficiency viruses. Our results suggest that BCA2 has different antiviral function between primate and non-primate retroviruses. In fact, the difference between non-primate lentiviruses and primate lentiviruses in the transmission process is also reflected in the host restriction factor SAMHD1 (Mereby et al., 2018). These results contribute to the understanding of the BCA2 antiviral mechanism and are helpful for studying the differences in host restriction factors in the transmission of primate or non-primate retroviruses.

## Materials and Methods

### Cell Cultures and Transfections

HEK293T cells were cultured and maintained in Dulbecco’s modified Eagle’s medium supplemented with 10% fetal bovine serum (Gibco) at 37°C and 5% CO_2_. Transfections of HEK293T cells were performed using Lipofectamine 2000 (Invitrogen) according to the manufacturer’s instructions.

### Plasmids

Total RNA was extracted from feline CrFK cells or HeLa cells using TRIzol (Invitrogen). The BCA2 sequences, including hBCA2, hBCA2 CA, hBCA2 KR, fBCA2, fBCA2 CA, and fBCA2 KR, were cloned into vector VR1012. HIV gag-HA and FIV gag-HA were codon-optimized and cloned into VR1012. All constructs were confirmed by sequencing. All primers were synthesized by the solid phase phosphoramidite triester method (Comate Bioscience, Jilin, China). The HIV-1 proviral clone pNL43, FIV pseudovirus clone (pFP93 and pGINSIN), FIV-14, pEGFP-N3, hBST2-HA, fBST2-HA, and VR1012 were described previously (Bisset et al., 2001; Lv et al., 2011; Wang et al., 2011; Wang et al., 2015).

### Antibodies

The following antibodies were used: anti-HA mouse monoclonal antibody (mAb) (Covance), anti-tubulin mouse mAb (Covance), anti-NF-κB 1 mouse mAb (Affinity Bioscience), anti-NF-κB p65 rabbit polyclonal antibody (Affinity Bioscience), anti-GAPDH rabbit polyclonal antibody (Affinity Bioscience), anti-Histone H3 rabbit polyclonal antibody (Proteintech), anti-Flag mouse mAb (Sigma), and anti-p24 mouse mAb obtained from an HIV-1 p24 hybridoma from the National Institutes of Health AIDS Research and Reference Reagent Program. Alkaline phosphatase-conjugated goat anti-rabbit or anti-mouse immunoglobulin G secondary antibodies were purchased from Jackson ImmunoResearch Laboratories (West Grove).

### Leupeptin, MG132, and Cycloheximide (CHX) Treatment

For lysosomal and proteasomal inhibition experiments, HEK293T cells were co-transfected with HIV-1 pNL43 and hBCA2-Flag, fBCA2-Flag, or VR1012. Twenty-four hours post-transfection, either DMSO, Leupeptin (20 μM) or MG132 (10 μM) was added to the medium. At 48 h after transfection, the lysates and released capsid proteins were analyzed by western blotting. For the CHX treatment experiment, HEK293T cells were co-transfected with HIV-1 Gag-HA plasmid along with hBCA2-Flag, fBCA2-Flag or VR1012. At 40 h after transfection, cells were treated with CHX (200 μg/mL) for different time. The cells were harvested and then analyzed by western blotting.

### Western Blotting

Cells were harvested at 48 h after transfection, washed once with pre-chilled PBS, and lysed in RIPA buffer for 20 min at 4°C, followed by addition of 4× SDS sample buffer. The samples were subjected to SDS-PAGE after boiling for 10 min, and the proteins were transferred onto nitrocellulose membranes by semi-dry transfer (Bio-Rad, Hercules, CA, United States). After blocking in 5% non-fat milk for 10 min, the membranes were probed with various primary antibodies against proteins of interest overnight at 4°C. After incubation with secondary antibodies, immunoreactions were visualized with 5-bromo-4-chloro-3 indolyl phosphate and nitro blue tetrazolium solutions.

### Nuclear and Cytoplasmic Fractionations

Five million HEK293T cells were transfected with 5 μg VR1012, hBCA2, or fBCA2. Forty-eight hours later, the cells were lysed and proteins were extracted to obtain cytoplasmic and nuclear extracts using the ProteoExtract^®^ Subcellular Proteome Extraction Kit (S-PEK), following the manufacturer’s instructions (Millipore, Bedford, MA, United States). The cytoplasmic fraction and nucleic protein fraction were analyzed by western blotting. Equal protein loading for cytoplasmic and nuclear fractions was confirmed by blotting against GAPDH.

### Co-immunoprecipitation Assay

At 48 h after transfection, 293 T cells were harvested, washed twice with cold PBS, and lysed in lysis buffer supplemented with Complete protease inhibitor cocktail tablets (EDTA Free, Roche, Basel, Switzerland) at 4°C for 45 min. The cell lysates were then centrifuged at 10,000 × *g* for 30 min, incubated with anti-Flag mAb at 4°C for 1.5 h and then incubated with protein G (Roche) at 4°C for 3 h. The beads were washed three times with washing buffer, boiled in SDS sample buffer, and subjected to SDS-PAGE.

### Virion Production

HIV-1 particles were produced by transient transfection of HEK293T cells in a 6-well plate with a proviral construct and indicated amounts of other plasmids. Forty-eight hours later, the supernatants (2 mL per culture) from producer cells were harvested, clarified by centrifugation, and passed through a 0.22 μm filter. The viral particles were concentrated through a 20% sucrose layer at 110,000 × *g* for 1.5 h and resuspended in 30 μL of RIPA buffer. Virus particle pellets and corresponding cell lysates were analyzed by SDS–PAGE and western blotting.

The FIV-ΔVif-ΔOrfA-ΔEnv-GFP reporter virus was produced by transfecting HEK293T cells with pFP93, pGINSIN, and pVSV-G at a mass ratio of 3:3:1. The supernatants were harvested, clarified and assayed for GFP fluorescence intensity using a fluorescence microplate reader to represent released virus yield. The cells also were harvested, and the fluorescence intensity was analyzed by flow cytometry to assess viral protein expression. The cells were analyzed on a BD Accuri^TM^ C6 Flow Cytometer (BD Biosciences, Franklin Lakes, NJ, United States).

### NF-κB Analyses

NF-κB activity was detected by luciferase assay. HEK293T cells maintained in 12-well plates were transfected with plasmids along with the pNF-κB-Luc reporter plasmid (Agilent Technologies, Santa Clara, CA, United States). Forty-eight hours later, the cells were disrupted in Triton X-100 lysis buffer. Lysates were transferred to a black flat bottom 96-well plate, and the Luciferase Assay System substrate (Promega Corporation, Madison, WI, United States) was added to the lysates according to the manufacturer’s instructions. Samples were analyzed in a Luminoskan Ascent microplate luminometer (Thermo Fisher Scientific).

### Transcription Analyses

HEK293T cells seeded in a 6-well plate were transfected with 1 μg of NL43 or FIV-14. Samples were collected at 6, 18, 24, or 48 h post-transfection. The cells were harvested, and total RNA was isolated and purified using TRIzol (Invitrogen). cDNA was synthesized from 1 μg of total RNA using a TransScript^TM^ All-in-One First-Strand cDNA Synthesis SuperMix for qPCR kit (TransGen Biotech, Beijing, China). For HIV-1 *nef* or FIV *pol* quantification (Bisset et al., 2001; Colomer-Lluch and Serra-Moreno, 2017), the SYBR green-based real-time qPCR (RT-qPCR) method was conducted using the TransStart Top Green qPCR SuperMix kit (TransGen Biotech) according to the manufacturer’s instructions. HIV-1 *nef* primers: (FW *nef* primer, 5’-GTA CCA GTT GAG CCA GAT AAG G-3’; RV *nef* primer, 5’-GCT GTC AAA CCT CCA CTC TAA C-3’) and FIV *pol* primers: (FW *pol* primer, 5’-CCA TTC CTC TTG ATC CAG ATT AT-3’; RV *pol* primer, 5’-AAA TCC AGC CTT GTG GTA GAC TAC A-3’). *nef* and *pol* mRNA levels were normalized to *GAPDH* mRNA to determine the relative expression ratio.

### Statistical Analysis

Group comparisons were performed using *t*-tests (and non-parametric tests). Calculations were performed with the INSTAT2 software package (GraphPad Software, San Diego, CA, United States). Results were given as the mean ± SEM. Statistical significance was accepted for *P* < 0.05.

## Data Availability Statement

Publicly available datasets were analyzed in this study. This data can be found here: 10.1371/journal.ppat.1000700, 10.1371/journal.ppat.1004151, and 10.1128/JVI.02098-16.

## Author Contributions

MQ, WW, and XY contributed to the conception of the study. MQ and WW designed and performed the experiments. WL, JC, and XZ assisted and performed the experiments. MQ, WW, CW, JW, BY, HZ, HW, WK, and XY contributed to analysis with constructive discussions. MQ, WW, and XY contributed significantly to the analysis of results and manuscript preparation. MQ, CW, and XY revised and edited the manuscript. All authors read and approved the final manuscript.

## Conflict of Interest

The authors declare that the research was conducted in the absence of any commercial or financial relationships that could be construed as a potential conflict of interest.
