# Supplementary material for: Antiviral Activity of Feline BCA2 Is Mainly Dependent on Its Interference With Proviral Transcription Rather Than Degradation of FIV Gag
Source: Front Microbiol. 2020 Jun 11;11:1230. doi: 10.3389/fmicb.2020.01230 (PMC7301684; doi:10.3389/fmicb.2020.01230)
Supplement: Supplementary file 1 [file Data_Sheet_1.docx]

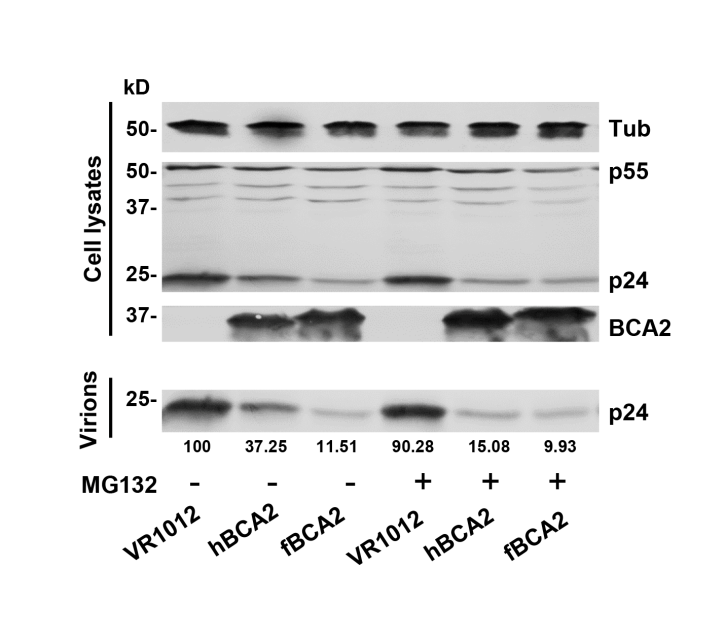


**FIGURE S1. Feline BCA2 does not promote proteasomal degradation of HIV-1.** HEK293T cells transfected with 1 μg of pNL43 and 500 ng of hBCA2-Flag, fBCA2-Flag or VR1012 were treated with 10 μM proteasomal inhibitor MG132 or DMSO as a control. MG132 was added to the medium at 24 h before harvesting. Cell lysates and supernatants were then analyzed by western blotting using anti-p24, anti-Flag, and anti-tubulin antibodies. Results of quantitative band scan analysis of the released virions are shown, and the percentage of VR1012 (DMSO) quantifications are set to 100%.
